# Supplementary material for: Sex and pressure effects of foam rolling on acute range of motion in the hamstring muscles
Source: PLoS One. 2025 Feb 24;20(2):e0319148. doi: 10.1371/journal.pone.0319148 (PMC11849903; doi:10.1371/journal.pone.0319148)
Supplement: Appendix 1 — (DOCX) [file pone.0319148.s001.docx]

| Appendix 1: Effect sizes of time-point differences in ROM of PSLR and PKE by sex and intensity levels | | | | | |
| --- | --- | --- | --- | --- | --- |
|  |  |  | Pre-Post | Pre-Post10 | Post-Post10 |
| PSLR | Female | CTRL | 0.19 | 0.05 | 0.05 |
|  |  | Low | 0.67 | 0.95 | 0.19 |
|  |  | High | 1.12 | 1.16 | 0.06 |
|  | Male | CTRL | 0.06 | 0.10 | 0.03 |
|  |  | Low | 0.37 | 0.32 | 0.05 |
|  |  | High | 0.54 | 0.69 | 0.16 |
| PKE | Female | CTRL | 0.25 | 0.02 | 0.21 |
|  |  | Low | 0.74 | 0.82 | 0.22 |
|  |  | High | 0.74 | 0.77 | 0.04 |
|  | Male | CTRL | 0.15 | 0.23 | 0.08 |
|  |  | Low | 0.65 | 0.54 | 0.13 |
|  |  | High | 0.49 | 0.54 | 0.16 |
