# Supplementary material for: Sex and pressure effects of foam rolling on acute range of motion in the hamstring muscles
Source: PLoS One. 2025 Feb 24;20(2):e0319148. doi: 10.1371/journal.pone.0319148 (PMC11849903; doi:10.1371/journal.pone.0319148)
Supplement: Appendix 2 — (DOCX) [file pone.0319148.s002.docx]

| Appendix 2: Effect sizes of intensity level comparisons in ROM of PSLR and PKE across time points by sex | | | | | |
| --- | --- | --- | --- | --- | --- |
|  |  |  | CTRL-Low | CTRL-High | Low-High |
| PSLR | Female | Pre | 0.19 | 0.21 | 0.01 |
|  |  | Post | 0.61 | 1.02 | 0.42 |
|  |  | Post10 | 0.87 | 1.03 | 0.25 |
|  | Male | Pre | 0.06 | 0.25 | 0.18 |
|  |  | Post | 0.39 | 0.35 | 0.05 |
|  |  | Post10 | 0.38 | 0.55 | 0.16 |
| PKE | Female | Pre | 0.44 | 0.29 | 0.15 |
|  |  | Post | 0.56 | 0.73 | 0.28 |
|  |  | Post10 | 0.44 | 0.53 | 0.10 |
|  | Male | Pre | 0.29 | 0.21 | 0.10 |
|  |  | Post | 0.55 | 0.42 | 0.23 |
|  |  | Post10 | 0.52 | 0.56 | 0.07 |
