# Supplementary material for: Sex and pressure effects of foam rolling on acute range of motion in the hamstring muscles
Source: PLoS One. 2025 Feb 24;20(2):e0319148. doi: 10.1371/journal.pone.0319148 (PMC11849903; doi:10.1371/journal.pone.0319148)
Supplement: Appendix 3 — (DOCX) [file pone.0319148.s003.docx]

| Appendix 3: Effect sizes of applied force differences across time points in PSLR and PKE by sex and intensity | | | | | |
| --- | --- | --- | --- | --- | --- |
|  |  |  | Pre-Post | Pre-Post10 | Post-Post10 |
| PSLR | Female | CTRL | 0.09 | 0.09 | 0.00 |
|  |  | Low | 0.30 | 0.18 | 0.13 |
|  |  | High | 0.45 | 0.40 | 0.06 |
|  | Male | CTRL | 0.04 | 0.28 | 0.25 |
|  |  | Low | 0.19 | 0.20 | 0.03 |
|  |  | High | 0.44 | 0.72 | 0.14 |
| PKE | Female | CTRL | 0.22 | 0.11 | 0.11 |
|  |  | Low | 0.44 | 0.92 | 0.38 |
|  |  | High | 0.22 | 0.21 | 0.00 |
|  | Male | CTRL | 0.06 | 0.30 | 0.33 |
|  |  | Low | 0.13 | 0.02 | 0.13 |
|  |  | High | 0.31 | 0.15 | 0.19 |
