# Supplementary material for: Sex and pressure effects of foam rolling on acute range of motion in the hamstring muscles
Source: PLoS One. 2025 Feb 24;20(2):e0319148. doi: 10.1371/journal.pone.0319148 (PMC11849903; doi:10.1371/journal.pone.0319148)
Supplement: Appendix 4 — (DOCX) [file pone.0319148.s004.docx]

| Appendix 4: Effect sizes of applied force comparisons across intensity levels in PSLR and PKE by sex and time points | | | | | |
| --- | --- | --- | --- | --- | --- |
|  |  |  | CTRL-Low | CTRL-High | Low-High |
| PSLR | Female | Pre | 0.08 | 0.09 | 0.00 |
|  |  | Post | 0.33 | 0.24 | 0.15 |
|  |  | Post10 | 0.21 | 0.21 | 0.05 |
|  | Male | Pre | 0.09 | 0.19 | 12.23 |
|  |  | Post | 0.30 | 0.26 | 0.04 |
|  |  | Post10 | 0.03 | 0.20 | 0.17 |
| PKE | Female | Pre | 0.20 | 0.03 | 0.22 |
|  |  | Post | 0.44 | 0.49 | 0.03 |
|  |  | Post10 | 0.78 | 0.33 | 0.45 |
|  | Male | Pre | 0.08 | 0.03 | 0.05 |
|  |  | Post | 0.02 | 0.18 | 0.24 |
|  |  | Post10 | 0.27 | 0.48 | 0.22 |
