# Supplementary material for: Sex and pressure effects of foam rolling on acute range of motion in the hamstring muscles
Source: PLoS One. 2025 Feb 24;20(2):e0319148. doi: 10.1371/journal.pone.0319148 (PMC11849903; doi:10.1371/journal.pone.0319148)
Supplement: Appendix 5 — (DOCX) [file pone.0319148.s005.docx]

| Appendix 5: Effect size of pain comparisons across intensity levels during FR intervention by sex | | | |
| --- | --- | --- | --- |
|  | CTRL-Low | CTRL-High | Low-High |
| Female | 0.07 | 1.38 | 1.53 |
| Male | 0.10 | 0.91 | 1.09 |
