# Supplementary material for: Sex and pressure effects of foam rolling on acute range of motion in the hamstring muscles
Source: PLoS One. 2025 Feb 24;20(2):e0319148. doi: 10.1371/journal.pone.0319148 (PMC11849903; doi:10.1371/journal.pone.0319148)
Supplement: Appendix 6 — (DOCX) [file pone.0319148.s006.docx]

| Appendix 6: Effect size of pain comparisons across time points during ROM measurements by sex and intensity levels | | | | |
| --- | --- | --- | --- | --- |
|  |  | Pre-Post | Pre-Post10 | Post-Post10 |
| Female | CTRL | 0.44 | 0.66 | 0.28 |
|  | Low | 0.19 | 0.35 | 0.22 |
|  | High | 0.05 | 0.29 | 0.34 |
| Male | CTRL | 0.16 | 0.06 | 0.25 |
|  | Low | 0.15 | 0.12 | 0.03 |
|  | High | 0.12 | 0.08 | 0.06 |
