# Supplementary material for: Sex and pressure effects of foam rolling on acute range of motion in the hamstring muscles
Source: PLoS One. 2025 Feb 24;20(2):e0319148. doi: 10.1371/journal.pone.0319148 (PMC11849903; doi:10.1371/journal.pone.0319148)
Supplement: Appendix 7 — (DOCX) [file pone.0319148.s007.docx]

| Appendix 7: Effect size of pain comparisons across intensity levels during ROM measurements by sex and time points | | | | |
| --- | --- | --- | --- | --- |
|  |  | CTRL-Low | CTRL-High | Low-High |
| Female | Pre | 0.33 | 0.37 | 0.05 |
|  | Post | 0.08 | 0.03 | 0.14 |
|  | Post10 | 0.04 | 0.73 | 0.71 |
| Male | Pre | 0.41 | 0.08 | 0.45 |
|  | Post | 0.47 | 0.03 | 0.43 |
|  | Post10 | 0.23 | 0.22 | 0.40 |
