# Supplementary material for: Sex and pressure effects of foam rolling on acute range of motion in the hamstring muscles
Source: PLoS One. 2025 Feb 24;20(2):e0319148. doi: 10.1371/journal.pone.0319148 (PMC11849903; doi:10.1371/journal.pone.0319148)
Supplement: Appendix 8 — (DOCX) [file pone.0319148.s008.docx]

| Appendix 8: Effect size of tightness comparisons across time points during ROM measurements by sex and intensity levels | | | | |
| --- | --- | --- | --- | --- |
|  |  | Pre-Post | Pre-Post10 | Post-Post10 |
| Female | CTRL | 0.01 | 0.26 | 0.30 |
|  | Low | 0.19 | 0.05 | 0.18 |
|  | High | 0.97 | 1.07 | 0.08 |
| Male | CTRL | 0.10 | 0.28 | 0.23 |
|  | Low | 0.57 | 0.43 | 0.17 |
|  | High | 0.51 | 0.54 | 0.03 |
