# Supplementary material for: Sex and pressure effects of foam rolling on acute range of motion in the hamstring muscles
Source: PLoS One. 2025 Feb 24;20(2):e0319148. doi: 10.1371/journal.pone.0319148 (PMC11849903; doi:10.1371/journal.pone.0319148)
Supplement: Appendix 9 — (DOCX) [file pone.0319148.s009.docx]

| Appendix 9: Effect size of tightness comparisons across intensity levels during ROM measurements by sex and time points | | | | |
| --- | --- | --- | --- | --- |
|  |  | CTRL-Low | CTRL-High | Low-High |
| Female | Pre | 0.10 | 0.28 | 0.38 |
|  | Post | 0.34 | 0.50 | 0.17 |
|  | Post10 | 0.51 | 0.93 | 0.41 |
| Male | Pre | 0.02 | 0.25 | 0.31 |
|  | Post | 0.53 | 0.05 | 0.52 |
|  | Post10 | 0.15 | 0.16 | 0.28 |
