# Supplementary material for: Sex and pressure effects of foam rolling on acute range of motion in the hamstring muscles
Source: PLoS One. 2025 Feb 24;20(2):e0319148. doi: 10.1371/journal.pone.0319148 (PMC11849903; doi:10.1371/journal.pone.0319148)
Supplement: Attachment — Submitted filename: Response to Reviewers.docx [file pone.0319148.s011.docx]

Reviewer #0

**Thank you very much for your thorough and thoughtful review of our manuscript. We sincerely appreciate the time and effort you have invested in providing detailed and constructive feedback. Your insightful comments have been invaluable in improving the quality and clarity of our work. We have carefully addressed all the points raised in your review and made the necessary revisions to the manuscript. A detailed response to each comment has been included in the letter for your reference. We hope that the revised manuscript meets your expectations and aligns with the high standards of the journal.**

Title and Abstract

Title: While the title reflects the content well, it includes technical language and could be simplified. A suggested revision might make the title more accessible to a broader readership.

Suggestion: A clearer title, such as “Gender and Pressure Effects of Foam Rolling on Acute Range of Motion in the Hamstring Muscles,” could be more understandable.

**Thank you for a valuable suggestion. We carefully considered the reviewer’s suggestion and modified the title to “Sex and Pressure Effects of Foam Rolling on Acute Range of Motion in the Hamstring Muscles"**

Abstract: According to PLOS ONE’s requirements, the abstract should be brief, clear, and highlight the main results. The current abstract contains excessive technical details, which may make it difficult for readers to grasp the key findings.

Suggestion: The abstract should be restructured to focus on the primary findings and significance of the study. For instance, numerical details like specific ROM values may overshadow the main findings and could be omitted.

**Thank you for a valuable suggestion. We revised the abstract to avoid redundant explanations about technical details.**

Introduction

Background Information: The introduction provides comprehensive information on the importance of ROM and foam rolling. However, some sentences are overly lengthy and complex, which hinders readability.

Suggestion: Simplify sentences such as "Foam rolling is a beneficial technique that increases ROM immediately after intervention without a reduced performance of sprint, jump, and strength" to improve clarity, e.g., “Foam rolling effectively increases range of motion without decreasing sprint, jump, or strength performance.”

**Thank you for your constructive feedback. We have revised the section to simplify overly complex sentences and improve clarity while retaining all essential information. We believe this adjustment enhances readability and aligns the content more closely with the reader’s expectations.**

Hypothesis and Aim: The study’s aim and hypothesis are clearly defined but could be emphasized further.

Suggestion: A more explicit statement such as, “The aim of this study is to investigate the acute effects of foam rolling on ROM based on gender and pressure” would enhance clarity.

**Thank you for your suggestion. We have revised the statement of the study’s aim and hypothesis to enhance clarity and emphasis, as suggested. The updated text provides a more explicit and straightforward description of the study’s purpose and hypothesis, aligning with the feedback to improve readability and focus. (L74-79)**

Materials and Methods

Ethics Statement and Participant Consent: According to PLOS ONE standards, studies involving human participants must clearly indicate ethics board approval and participant consent. While the ethics approval number is provided, further detail on the approval process is lacking. Per PLOS ONE guidelines, the study should state that it was conducted in accordance with the Declaration of Helsinki and that written consent was obtained from all participants.

Suggestion: Include a statement such as, “This study was conducted in accordance with the Declaration of Helsinki and approved by the Ethics Committee of XXXXX University (approval number: 2020-418). Written consent was obtained from all participants.”

**Thank you very much for your valuable comment. In accordance with all the reviewers’ comments, we revised more detail as follows.**

*L102-109: This study was conducted in full compliance with the Declaration of Helsinki. Ethical approval was obtained from the Ethics Committee of Waseda University, approval number 2020-418. All participants were provided comprehensive information regarding the study objectives, procedures, potential risks, and benefits. Written informed consent was obtained from each participant prior to their involvement in the study. Participants were informed that their participation was voluntary and that they could withdraw from the study at any time without any repercussions. Furthermore, all data collected were anonymized to ensure participant confidentiality.*

Data Accessibility: PLOS ONE requires clear accessibility of research findings and data, especially for reuse purposes. It is stated that the data are stored in a third-party repository, but the access details and DOI are unclear.

Suggestion: Add a statement like, “All data supporting this study’s findings are publicly available in the Dryad Digital Repository (DOI: https://doi.org/10.5061/dryad.1ns1rn93x).” (Minor Revision)

**Thank you for your comment. We revised it following the reviewer’s comment. The submitted data will be available after the curation process by Dryad.**

Measurement Methods: While the methods for measuring ROM and strength are described in detail, there is a lack of information regarding the calibration and reliability of the equipment used. No details are provided about the calibration of devices such as the force plate and hand dynamometer.

Suggestion: Add a statement like, “Regular calibration was conducted to ensure the reliability of devices used for ROM and strength measurements.” (Minor Revision)

**Thank you for your valuable comment. We agree that including information about the calibration and reliability of the equipment is essential for transparency and rigor. We have revised the Methods section to include the following statement:**

*L133-135: Before the experiment, regular calibration was conducted to ensure the reliability of devices used for ROM and strength measurements, including the force plate and hand dynamometer.*

Statistical Analyses: Although the statistical analyses are generally well-explained, PLOS ONE guidelines recommend reporting effect sizes alongside significance tests. ANOVA and post-hoc tests were used, but effect sizes (e.g., partial eta-squared) were not reported.

Suggestion: Report effect sizes, such as η² or Cohen’s d, alongside significance values in the statistical analysis. (Minor Revision)

**Thank you for your insightful comment. We have revised the Statistical Analyses section to include effect sizes (e.g., Cohen’s d) alongside significance tests. For post-hoc comparisons, effect sizes are reported for all pairs where significant differences were observed after Bonferroni correction. Additionally, for completeness, all effect sizes, including those for non-significant comparisons, are presented in the Appendix to provide a comprehensive overview of the results. In the main text, only the range of effect sizes for post-hoc comparisons with statistically significant differences is reported. Moreover, partial η² values for the ANOVA interactions are now included in the table footnotes to provide further context regarding the magnitude of effects observed in the study. We also acknowledge that the use of partial η² for ANOVA was not initially mentioned in the Methods section. This has now been rectified, ensuring consistency between the Methods and Results sections.**

*L203-208: Effect sizes for the ANOVA and post hoc tests were assessed using partial η² and Cohen’s d. Thresholds for partial η² were set as small (0.01), medium (0.06), and large (0.14)[28]. For Cohen’s d, the thresholds were small (0.20), medium (0.50), and large (0.80)[28].*

**In response, we have revised the relevant section to incorporate the finding that female participants demonstrated a larger effect size compared to males, while emphasizing that no statistically significant difference was observed. The revised sentence is as follows:**

*L313-319: In contrast to our hypothesis, the sex difference in FR-induced acute improvement in ROM was not statistically significant. While female participants showed a larger effect size compared to males, the results do not sufficiently support the suggestion of a previous study that female participants are more sensitive to FR interventions*

Results

Tables and Figures: The results section includes numerous tables and detailed data, but some tables may hinder readers from understanding the findings clearly.

Suggestion: Simplify tables to focus on key findings. For instance, general trends by gender and pressure could be summarized, focusing on tables that present significant statistical differences. (Minor Revision) Clarity of Statistical Findings: Excessive numerical details in the results may obscure the main findings.

Suggestion: Emphasize the main findings (e.g., differences in ROM by gender and pressure) and avoid excessive detail on minor subgroup analyses. (Minor Revision)

**Thank you for your comments. To improve clarity and focus on the main findings, we revised the results section, including tables, by simplifying the description and emphasizing the significant interactions and key differences. The revised text maintains the statistical rigor while making the findings more accessible to readers.**

Discussion

Interpretation and Significance of Findings: The discussion addresses the study’s findings thoroughly, but the broader implications and practical applications are not clearly conveyed.

Example Sentence: "The acute changes in ROM observed in this study were comparable with those reported in previous studies.”

Suggestion: To provide broader context, consider a statement like, “The acute ROM changes observed in this study have potential benefits for enhancing mobility in athletes and physical therapy patients.” (Minor Revision)

**Thank you for your insightful comment. We have revised the discussion to better convey the broader implications and practical applications of our findings. The updated text emphasizes that the use of readily available tools, such as a soccer ball, can achieve similar ROM benefits to those obtained with specialized equipment. This revision highlights the potential applicability of our findings to athletes and physical therapy patients who may lack access to specialized devices.**

*L250-252: The acute ROM changes observed in this study were comparable with those reported in previous studies using specialized equipment. This finding highlights the potential of using readily available tools, such as a soccer ball, to achieve similar mobility benefits. Such an approach could be particularly beneficial for athletes and physical therapy patients who may not have access to specialized FR equipment.*

Limitations and Future Research: The limitations of the study (e.g., the use of two different pressure applications in a single day) are well-explained, but more specific suggestions for future research could be provided.

Suggestion: Add concrete suggestions, such as, “Future studies could explore the effects of different foam rolling devices or include larger and more diverse participant groups.” (Minor Revision)

**Thank you for your thoughtful comment. We have revised the Limitations section to include more concrete suggestions for future research, such as separating pressure conditions across different days, employing more precise monitoring systems, and including more diverse participant groups. These additions aim to address the study's limitations and provide a clearer direction for future studies. (L333-348)**

Reviewer #1:

**Thank you very much for your thorough and thoughtful review of our manuscript. We sincerely appreciate the time and effort you have invested in providing detailed and constructive feedback. Your insightful comments have been invaluable in improving the quality and clarity of our work. We have carefully addressed all the points raised in your review and made the necessary revisions to the manuscript. A detailed response to each comment has been included in the letter for your reference. We hope that the revised manuscript meets your expectations and aligns with the high standards of the journal.**

Title and Abstract: Restructuring the title and abstract to present the main findings in a more straightforward manner could facilitate quick comprehension for readers. In particular, reducing technical details in the abstract to highlight general findings and practical applications would better convey the significance of the study.

**Thank you for a valuable suggestion. We modified the title to “Sex and Pressure Effects of Foam Rolling on Acute Range of Motion in the Hamstring Muscles” in accordance with another reviewer's suggestion. We also edited the abstract to avoid redundant explanations about technical details.**

Ethics and Data Access: A more detailed ethical statement in accordance with the Declaration of Helsinki and a clarification of data accessibility are recommended. Such additions will enhance the study's credibility and allow readers the option to verify findings.

**Thank you for your suggestion. We explain a more detailed ethical statement as follows:**

*L102-109: This study was conducted in full compliance with the Declaration of Helsinki. Ethical approval was obtained from the Ethics Committee of Waseda University, approval number 2020-418. All participants were provided comprehensive information regarding the study objectives, procedures, potential risks, and benefits. Written informed consent was obtained from each participant prior to their involvement in the study. Participants were informed that their participation was voluntary and that they could withdraw from the study at any time without any repercussions. Furthermore, all data collected were anonymized to ensure participant confidentiality.*

Presentation of Results: Simplifying tables and graphics and focusing on key findings may improve the readability of the results. Emphasizing primary findings through visuals will help readers grasp the study’s implications more efficiently.

**Thank you for your comments. To improve clarity and focus on the main findings, we revised the results section by simplifying the description and emphasizing the significant interactions and key differences. The revised text maintains the statistical rigor while making the findings more accessible to readers.**

Future Research and Limitations: It is suggested to expand the discussion to include the study’s potential applications and future research directions. Clearly addressing limitations and providing concrete suggestions for future studies would enhance the study’s academic value.

**Thank you for your thoughtful comment. We have expanded the discussion to include the study’s potential applications and future research directions. The revised section provides a clearer outline of the study's limitations and includes concrete suggestions for addressing them in future studies. Additionally, we have highlighted the practical implications of our findings to enhance the study's academic and practical value. (L313-332)**

Reviewer #2:

**Thank you very much for your thorough and thoughtful review of our manuscript. We sincerely appreciate the time and effort you have invested in providing detailed and constructive feedback. Your insightful comments have been invaluable in improving the quality and clarity of our work. We have carefully addressed all the points raised in your review and made the necessary revisions to the manuscript. A detailed response to each comment has been included in the letter for your reference. We hope that the revised manuscript meets your expectations and aligns with the high standards of the journal.**

Line 42: Can you check the citations here. You have mentioned here that FR increases ROM but I do have some concerns regarding the references you have here. I just checked some of them and saw some confusion. In citation 1, there are findings favoring foam rolling group in jump, agility, bench press and sprint, so, not only in sprint. Furthermore, I suggest you to consider Mikesky’s study cautiously because the focus is not ROM in their study, there is no performance improvement in any parameter, and also they do not have much data regarding ROM to discuss. It is similar to reference number 3. There is no performance difference, yet there are some parameters such as fatigue. Also, ROM is not a focus in this study either. You should also consider the results in reference number 5. There are different vertical jump findings in SMR_5 and SMR_1 conditions. You have already mentioned “1-2 min interventions” in your paper but may be adding one or two sentence to explain time-related issues while applying FR for different performance parameters might inform the readers better in introduction part. Overall, can you please reconsider and confirm whether these references suggest FR increases ROM cause this is what you are saying in the core of this sentence.

**Thank you very much for the reviewer’s insightful comment. We carefully consider the comment, and revised the sentence to unable readers to understand the importance of FR for conditioning and rehabilitation through acute ROM improvement as follows:**

*L34-37: Improving the range of motion (ROM) before any exercise in athletes and patients is a frequent goal in conditioning and rehabilitation, where foam rolling (FR) is widely used. FR is a beneficial technique that increases ROM immediately after intervention [1], without a reduced performance of jump and strength [2].*

Line 48-51: Needs rewriting. I guess you are trying to say that the effects observed in studies including only men are smaller than the ones including females or mixed samples. Then, say it simply. You can also divide the sentence into two in order to make it easier to follow and understand for all.

**Thank you for your comment. We revised the sentence to give everyone a better understanding as follows:**

*L42-46: A recent meta-analysis found that studies including only male participants showed smaller changes in ROM after FR interventions compared to studies including females or mixed-sex samples. This suggests that ROM improvements induced by FR may be less pronounced in males. However, there is no consensus on this issue, highlighting the need for further empirical research to clarify these findings [1].*

Line 77-78: Needs rewriting.

**Thank you for your comment. We revised it to a more transparent and more precise explanation as follows;**

*L69-72: While pain is not always a prerequisite for FR’s effectiveness [21], the intensity of the mechanical stimuli may have a limited impact on outcomes [21,22]. This suggests that rolling and pressure stimulation are critical for acute ROM improvements, regardless of equipment type or intensity.*

Line 100-101: Can you divide the sentence into two to make it easier to follow as “… athletes training for at least 3 days a week . Our exclusion criteria were: current injury ….” ?

**Thank you for your suggestions. We revised the sentences following the reviewer’s comment.**

*L93-96: We recruited collegiate athletes who trained for at least 3 days per week. Our exclusion criteria were: current injuries, a history of severe injury in the lower extremity or lower back, and muscle soreness prior to the experiment.*

Line 103: Can you change it to: A total of 20 athletes (10 female and 10 male) participated …

**Thank you for your comment. We revised it following the reviewer’s comment. (L97)**

Line 105: Can you change it to: They were asked not to engage in exhausting activity….

**Thank you for your comment. We revised it following the reviewer’s comment.**

Line 109: Approval number: 2020-418

**Thank you for your comment. We revised the sentence totally. Please re-check the sentence. (L103-104)**

For participants, G*Power analysis suggested 40 participants but you only included 20? The authors explained the study protocol in detail.

**Thank you for your comment. First of all, please allow me to correct the information in the relevant section, as there was an error in the original description. Including the control condition, the intensity of pressure consists of three levels, making the between-factor design 6 conditions (2 sexes × 3 intensities). Upon recalculating the minimum required sample size under these conditions, the result indicates 5 participants per group, which corresponds to 5 participants for each sex. However, considering the uncertainties in effect size, adjustments for non-sphericity, as well as the influence of individual differences and measurement errors, it is deemed more appropriate to include 10 participants per group. Therefore, the following adjustments and corrections have been made accordingly.**

*L82-93: The calculation for the a priori F test with analysis of variance (ANOVA) repeated measures, within-between interaction of six groups (two sexes and three pressures) and three measurements (three time points), was conducted, given an effect size of 0.25, an α error probability of 0.05, and a power of 0.80, with a correlation among repeated measures of 0.5 and a non-sphericity correction (ε) of 1. The total required sample size was determined to be 30 for six groups, with five participants per group, which corresponds to five participants for each sex as the minimum sample size. However, considering the need to reduce the influence of individual differences and measurement errors, to address uncertainties in effect size, to mitigate the impact of missing data and adjustments for non-sphericity, and to enhance the reproducibility of the experimental results, the study included 10 participants per group, resulting in a total of 20 participants for each sex.*

Line 184: … ”almost perfect” as in a previous …

**Thank you for your comment. We revised it following the reviewer’s comment. (L183)**

In the whole text, for the word “pain” that you have evaluated, it is better to use “perceived pain” because you just ask participants regarding their thoughts and perceptions. So, can you change them into “perceived pain”?

**Thank you very much for your comment. We fully agree with the reviewer’s suggestion. The term "pain" is initially introduced as "perceived pain" (hereafter referred to as "pain") and is consistently used as "pain" throughout the method and results section. However, in the discussion section, all references to "pain" will be written as "perceived pain" instead. Please let us know if further revisions are required.**

Line 202: The perceived pain in the following morning was investigated…

**Thank you for your comment. We revised it following the reviewer’s comment. (L191, 201)**

Line 204: No need for that sentence: “Statistical significance was set at p<0.05.” Because you also explain it in the next lines.

**Thank you for your comment. We deleted it following the reviewer’s comment.**

Line 226: .. in the following morning.

**Thank you for your comment. We revised it following the reviewer’s comment. (L232)**

All tables were prepared good and explained well in the comments in results section.

**Thank you for your comment. We have revised it in accordance with the other reviewer’s comment. Please re-check them.**

Line 243: … hamstring muscles using commercially available soccer balls improved…

**Thank you for your comment. We revised it following the reviewer’s comment. (L249)**

Line 250-254: Can you please check the article you have cited here (number 21)? Can you check the numbers and the other details?

**Thank you very much for your comment. We mistakenly attributed the citation to the wrong section. Morales et al. (2017) demonstrated that FR intervention alone did not result in changes in the PKE ROM of male participants. In contrast, this sentence highlights that ROM changes were observed in male participants in this study following the FR intervention. We revised the sentence as follows;**

*L260-264: In a previous study, while no significant acute improvement was observed in chair-seated passive knee extension at 5 min after FR intervention (approximately 1.5%)[1]7, male participants of this study demonstrated substantial acute improvement of PKE in 5.4% (from 143.2±4.1° to 150.9±3.2°) under low pressure, and 3.9% (144.8±3.4° to 150.5±3.6°) under high pressure 10 min following intervention.*

Line 258: Instead of device, use tool or equipment. (You have used “equipment” in the following sections, so changing it to “equipment” might work better, but you can decide. Just make sure that it is coherent with the following statements as well.)

**Thank you for your comment. We revised to equipment instead of device. (L268)**

Line 260: Check the cited articles (36-38) whether they really say so.

**Thank you for your insightful comment. Upon reviewing the cited articles, we found that only Paoloni et al. directly addressed manual massage and its effects on muscle extensibility. While Paoloni et al. did not explicitly investigate ROM changes, their findings suggest that enhancing muscle extensibility through manual therapy, including massage, may contribute to ROM improvements. Therefore, we revised the statement to exclusively cite Paoloni et al. and removed the references to Schroeders et al. and Schleip et al., as their studies primarily discuss self-myofascial release or pressure stimulation without directly addressing manual massage. This adjustment ensures the accuracy and relevance of our citations. We revised the corresponding sentence as follows:**

*L268-270: Manual massage, which involves physical pressure stimulation of the skin, has been shown to enhance muscle extensibility, which may contribute to changes in ROM [30].*

Line 266: Use the citation below instead of both studies (13,9) because they also cited this article while discussing about higher blood flow. They do not have any data showing the case for higher blood flow but Hotfiel et al does! I suggest you to go to the original study. I think it is more ethical to give the credits to those who conduct the original study instead of citing others.

Hotfiel T, Swoboda B, Krinner S, Grim C, Engelhardt M, Uder M, et al. Acute effects of lateral thigh foam rolling on arterial tissue perfusion determined by spectral doppler and power doppler ultrasound. J Strength Cond Res. 2017;31(4):893–900.

**Thank you for your comment. We have revised it in accordance with the other reviewer’s comment. (L276)**

Line 266-270: Needs rewriting.

**Thank you for your insightful comment. We carefully reviewed the cited content and original statement to ensure accuracy and clarity. Upon review, we recognized that the original phrasing could be refined to better align with the findings and implications discussed in the cited study.**

*L277-283: The rolling on the skin, fascia, and muscle generates friction, which may increase tissue temperature and potentially enhance range of motion (ROM). Additionally, rolling may apply shear stress to tissues, reducing intra- and inter-cellular fluid viscosity and making movement less resistant. It is plausible that these mechanical effects, including friction and shear stress, contribute to temporary changes in ROM. These findings underscore the potential of physical stimuli, such as pressure and rolling, to influence ROM [33].*

Line 270-281: I do not think you need to give that much place for such discussions because it is not a part of your study, you do not have any sympathetic activity data. Maybe, you can just mention it in a sentence and go through your main focus and data-related discussions.

**Thank you for your comment. We have simplified the discussion on sympathetic nerve activity and its relationship with ROM to maintain the focus on our study's primary findings. The revised text now briefly mentions this mechanism as a potential pathway without delving into details. If we need to delete all these sentences, we will re-consider it.**

*L283-285: FR interventions may acutely improve ROM through mechanisms such as muscle relaxation and possible changes in sympathetic nerve activity [11,12], though further research is needed to confirm these pathways.*

Line 288: Can you check your statement here and your citation. Can you confirm Wilke et al has any related part in their article saying hamstring is more sensitive to FR than quadriceps? On the contrary, there is a part in Wilke et al saying:

**Thank you for your comment. Upon reviewing the article by Wilke et al., we acknowledge that their findings do not explicitly state that the hamstring muscles are more sensitive to foam rolling than the quadriceps muscles. Instead, their study reported that foam rolling significantly increased ROM in the hamstrings compared to the calf muscles, while the effects on the quadriceps did not reach statistical significance due to data variability and broad confidence intervals. Based on this, we revised the statement as follows:**

*L289-292: The hamstring muscles may respond more noticeably to FR than the calf muscles, while quadriceps effects were inconclusive due to data variability [1], while pressure magnitude has limited impact on acute ROM changes.*

Line 305: Correct the citation as Aboodarda et al. [14]… .

Line 319: Correct the citation as Arendt-Nielsen et al. [number]…

**Thank you for your comment. We revised it following the reviewer’s comment. (L308, 323)**

Line 342: “Vital” is not a good word choice. They are just two options along with some other ways. So, you can only say your findings suggested that applying pressure and rolling stimuli on targeted muscles are good ways to improve acute ROM changes … .

**Thank you for your comment. We revised it to “effective method” instead of vital. (L360-361) In response, we revised “vital” to “crucial” in L267.**

In conclusion part, you can add similar sentence to the sentence you have in line 325-326 (further research is needed regarding sex differences in FR-induced acute ROM changes by adopting various FR conditions such as speed, duration, and surface of the equipment to modulate stimulation) to highlight the need for considering different moderators.

**Thank you for your suggestion. We have revised the conclusion to include a statement emphasizing the need for further research on sex differences and the exploration of diverse FR conditions, as mentioned in lines 325-326. This addition highlights the importance of considering different moderators to better understand and optimize FR interventions.**

*L360-369: Applied pressure and rolling stimuli on targeted muscles are effective methods for improving acute ROM changes, regardless of sex. While FR pressure magnitude and the degree of perceived pain may not strongly affect acute ROM changes, the effective conditions for inducing such changes appear to have greater individual specificity than previously recognized. Therefore, further research is needed to identify individual effective conditions, particularly by exploring diverse attributes of stimuli, such as rolling speed, duration, and the surface texture of the equipment. Additionally, investigations into sex differences in FR-induced acute ROM changes under varying FR conditions will provide valuable insights into personalized intervention strategies.*

In response to the revision, we deleted and added several references as follows:

Deleted

- Peacock CA, Krein DD, Silver TA. An acute bout of self-myofascial release in the form of foam rolling. Int J Exerc Sci. 2014;7: 202–211.
- Mikesky AE, Bahamonde RE, Stanton K, Alvey T, Fitton T. Acute effects of the stick on strength, power, and flexibility. J Strength Cond Res. 2002;16: 446–450.
- Healey KC, Hatfield DL, Blanpied P, Dorfman LR, Riebe D. The effects of myofascial release with foam rolling on performance. J Strength Cond Res. 2014;28: 61–68.
- Phillips J, Diggin D, King DL, Sforzo GA. Effect of varying self-myofascial release duration on subsequent athletic performance. J Strength Cond Res. 2021;35: 746–753.
- McGlone F, Reilly D. The cutaneous sensory system. Neuroscience and Biobehavioral Reviews. 2010. pp. 148–159. doi:10.1016/j.neubiorev.2009.08.004
- Hubbard T. Review of myofascial release as an effective massage therapy technique. Athletic Therapy Today. 2009;14: 30–34.
- Schleip R, Müller DG. Training principles for fascial connective tissues: Scientific foundation and suggested practical applications. J Bodyw Mov Ther. 2013;17: 103–115. doi:10.1016/j.jbmt.2012.06.007
- Schroeder AN, Best TM. Is self myofascial release an effective preexercise and recovery strategy? A literature review. 2015. Available: <http://journals.lww.com/acsm-csmr>
- Etzi R, Carta C, Gallace A. Stroking and tapping the skin: behavioral and electrodermal effects. Exp Brain Res. 2018;236: 453–461. doi:10.1007/s00221-017-5143-9
- Roatta S, Farina D. Sympathetic actions on the skeletal muscle. Exerc Sport Sci Rev. 2010. Available: [www.acsm-essr.org](http://www.acsm-essr.org)
- Guimaraes S, Moura D. Vascular adrenoreceptors: An update. Pharmacol Rev. 2001;53: 319–356.

Added

- Nakamura M, Onuma R, Kiyono R, Yasaka K, Sato S, Yahata K, et al. The acute and prolonged effects of different durations of foam rolling on range of motion, muscle stiffness, and muscle strength. J Sports Sci Med. 2021;20: 62–68. doi:10.52082/jssm.2021.62
- Paoloni J. Review of myofascial release as an effective massage therapy technique. Athletic Therapy Today. 2009;14: 30–34.
- Cohen J. Statistical power analysis for the behavioral sciences. 2nd ed. London: Routledge; 1988.
