# Supplementary material for: Sex and pressure effects of foam rolling on acute range of motion in the hamstring muscles
Source: PLoS One. 2025 Feb 24;20(2):e0319148. doi: 10.1371/journal.pone.0319148 (PMC11849903; doi:10.1371/journal.pone.0319148)
Supplement: Attachment — Submitted filename: Reviewer Comments.docx [file pone.0319148.s010.docx]

**Title and Abstract**

**Title:** While the title reflects the content well, it includes technical language and could be simplified. A suggested revision might make the title more accessible to a broader readership.

**Suggestion:** A clearer title, such as “Gender and Pressure Effects of Foam Rolling on Acute Range of Motion in the Hamstring Muscles,” could be more understandable.

**Abstract:** According to PLOS ONE’s requirements, the abstract should be brief, clear, and highlight the main results. The current abstract contains excessive technical details, which may make it difficult for readers to grasp the key findings.

**Suggestion:** The abstract should be restructured to focus on the primary findings and significance of the study. For instance, numerical details like specific ROM values may overshadow the main findings and could be omitted.

**Introduction**

**Background Information**: The introduction provides comprehensive information on the importance of ROM and foam rolling. However, some sentences are overly lengthy and complex, which hinders readability.

**Suggestion:** Simplify sentences such as "Foam rolling is a beneficial technique that increases ROM immediately after intervention without a reduced performance of sprint, jump, and strength" to improve clarity, e.g., “Foam rolling effectively increases range of motion without decreasing sprint, jump, or strength performance.”

**Hypothesis and Aim:** The study’s aim and hypothesis are clearly defined but could be emphasized further.

**Suggestion:** A more explicit statement such as, “The aim of this study is to investigate the acute effects of foam rolling on ROM based on gender and pressure” would enhance clarity.

**Materials and Methods**

**Ethics Statement and Participant Consent:** According to PLOS ONE standards, studies involving human participants must clearly indicate ethics board approval and participant consent.

While the ethics approval number is provided, further detail on the approval process is lacking. Per PLOS ONE guidelines, the study should state that it was conducted in accordance with the Declaration of Helsinki and that written consent was obtained from all participants.

**Suggestion:** Include a statement such as, “This study was conducted in accordance with the Declaration of Helsinki and approved by the Ethics Committee of XXXXX University (approval number: 2020-418). Written consent was obtained from all participants.”

**Data Accessibility:** PLOS ONE requires clear accessibility of research findings and data, especially for reuse purposes.

It is stated that the data are stored in a third-party repository, but the access details and DOI are unclear.

**Suggestion:** Add a statement like, “All data supporting this study’s findings are publicly available in the Dryad Digital Repository (DOI: https://doi.org/10.5061/dryad.1ns1rn93x).” (Minor Revision)

**Measurement Methods**: While the methods for measuring ROM and strength are described in detail, there is a lack of information regarding the calibration and reliability of the equipment used.

No details are provided about the calibration of devices such as the force plate and hand dynamometer.

**Suggestion:** Add a statement like, “Regular calibration was conducted to ensure the reliability of devices used for ROM and strength measurements.” (Minor Revision)

**Statistical Analyses:** Although the statistical analyses are generally well-explained, PLOS ONE guidelines recommend reporting effect sizes alongside significance tests.

ANOVA and post-hoc tests were used, but effect sizes (e.g., partial eta-squared) are not reported.

**Suggestion:** Report effect sizes, such as η² or Cohen’s d, alongside significance values in the statistical analysis. (Minor Revision)

**Results**

**Tables and Figures:** The results section includes numerous tables and detailed data, but some tables may hinder readers from understanding the findings clearly.

**Suggestion:** Simplify tables to focus on key findings. For instance, general trends by gender and pressure could be summarized, focusing on tables that present significant statistical differences. (Minor Revision)

**Clarity of Statistical Findings:** Excessive numerical details in the results may obscure the main findings.

**Suggestion:** Emphasize the main findings (e.g., differences in ROM by gender and pressure) and avoid excessive detail on minor subgroup analyses. (Minor Revision)

Discussion

**Interpretation and Significance of Findings**: The discussion addresses the study’s findings thoroughly, but the broader implications and practical applications are not clearly conveyed.

**Example Sentence:** "The acute changes in ROM observed in this study were comparable with those reported in previous studies.”

**Suggestion:** To provide broader context, consider a statement like, “The acute ROM changes observed in this study have potential benefits for enhancing mobility in athletes and physical therapy patients.” (Minor Revision)

**Limitations and Future Research:** The limitations of the study (e.g., the use of two different pressure applications in a single day) are well-explained, but more specific suggestions for future research could be provided.

**Suggestion:** Add concrete suggestions, such as, “Future studies could explore the effects of different foam rolling devices or include larger and more diverse participant groups.” (Minor Revision)

***Overall Evaluation***

*The study is well-structured and rigorously executed, but some sections are overly technical and contain details that may hinder quick comprehension.*

***General Recommendation:*** *Addressing minor revisions, particularly by including effect sizes in statistical analyses and enhancing the ethics and data accessibility statements, will make the article more understandable and better aligned with journal requirements.*
